# Shift Work and Obesity among Canadian Women: A Cross-Sectional Study Using a Novel Exposure Assessment Tool

**DOI:** 10.1371/journal.pone.0137561

**Published:** 2015-09-16

**Authors:** Natalie McGlynn, Victoria A. Kirsh, Michelle Cotterchio, M. Anne Harris, Victoria Nadalin, Nancy Kreiger

**Affiliations:** 1 Surveillance and Epidemiology Unit, Performance and Standards, Toronto Public Health, Toronto, Ontario, Canada; 2 Dalla Lana School of Public Health, University of Toronto, Toronto, Ontario, Canada; 3 Prevention and Cancer Control, Cancer Care Ontario, Toronto, Ontario, Canada; 4 School of Occupational and Public Health, Ryerson University, Toronto, Ontario, Canada; Institute for Health & the Environment, UNITED STATES

## Abstract

**Background/Objectives:**

It has been suggested that the association between shift work and chronic disease is mediated by an increase in obesity. However, investigations of the relationship between shift work and obesity reveal mixed findings. Using a recently developed exposure assessment tool, this study examined the association between shift work and obesity among Canadian women from two studies: a cohort of university alumni, and a population-based study.

**Methods:**

Self-administered questionnaire data were used from healthy, currently employed females in a population-based study, the Ontario Women’s Diet and Health case-control study (n = 1611 controls), and from a subset of a of university alumni from the Canadian Study of Diet, Lifestyle, and Health (n = 1097) cohort study. Overweight was defined as BMI≥25 to <30, and obesity as BMI≥30. Reported occupation was converted to occupational codes and linked to a probability of shift work value derived from Survey of Labour and Income Dynamics data. Regular evenings, nights, or rotating work comprised shift work. Polytomous logistic regression estimated the association between probability of shift work, categorized as near nil, low, medium, and high probability of shift work, on overweight and obesity, controlling for detected confounders.

**Results:**

In the population-based sample, high probability of shift work was associated with obesity (reference = near nil probability of shift work, OR: 1.88, 95% CI: 1.01–3.51, *p* = 0.047). In the alumni cohort, no significant association was detected between shift work and overweight or obesity.

**Conclusions:**

As these analyses found a positive association between high probability of shift work exposure and obesity in a population-based sample, but not in an alumni cohort, it is suggested that the relationship between shift work and obesity is complex, and may be particularly susceptible to occupational and education-related factors within a given population.

## Introduction

Shift work entailing circadian disruption is associated with workplace injury[1] and chronic disease.[2] Globally, work outside a conventional daytime schedule is not uncommon. In Canada, 20–30% of workers can be classified as shift workers,[3,4] and 13% of employed Canadians work regular night or rotating shifts exclusively.[5] Although necessary to meet a constant demand for around-the-clock labour, shift workers are at an increased risk for Type II diabetes,[6] various cancers,[7,8] heart disease and cardiovascular events including stroke.[9]

Other risk factors for the same morbidities associated with shift work include obesity and overweight.[10,11] In Canada, 62% of adults are above normal weight, and 1 in 4 are obese.[12,13] Obesity has strong associations with several debilitating and life-threatening health conditions, and can become a chronic condition in itself, as losing weight and maintaining weight loss becomes increasingly difficult with increases in body mass.[12–14] Once considered a disease unique to the wealthy, the prevalence of obesity has risen in both developed and developing countries, across socio-economic groups, and is now considered a global epidemic and a leading cause of death worldwide.[11,15,16]

Obesity is a complex result of a variety of factors.[16,17] Recently, shift work has been implicated in weight gain, and it has been suggested that obesity mediates the relationship between shift work and various morbidities.[18,19] Theories posit that shift work promotes weight gain through behavioural dysregulation, such as a lack of time to exercise, in addition to hormonal and diet factors related to circadian rhythm disruption and sleep deprivation.[2] However, investigations of the relationship between shift work and obesity report mixed findings.[20] Many of these studies have detected an increased BMI, or more overweight or obesity amongst shift workers compared to day workers in at least one analysis group.[20–25]. Other studies, however, failed to find any association [26–29] and at least one study detected a negative association between unconventional work hours and weight.[30] Furthermore, most studies that have explored the relationship between shift work and obesity have focused on blue-collar workers or nurses, with relatively few studies exploring the relationship within a mixed cohort of workers.[20]

More research is needed in order to better understand the association between shift work and obesity. In Canada, only two studies of which we are aware have been conducted in this area. In one study, shift work was not associated with unhealthy weight gain among Canadian males and females.[31] In the other study, a higher BMI was detected among female nurses who work shifts, with no differences in BMI according to shift schedule detected for male nurses, although the male sample was smaller.[24]

The aim of our study was to investigate the association between shift work and obesity among Canadian females from a variety of occupations. Since no previous study of which we are aware has analyzed the relationship between shift work and obesity within a diversified workforce that is highly educated, we investigated the relationship within a Canadian cohort of university alumni alongside a population-based sample of healthy controls originally collected as a case-control study. We determined shift work exposure through a recently created exposure assessment derived from Canadian Survey of Labour and Income Dynamics (SLID) data.[32]

## Methods

### Sample and Data Source

This is a cross-sectional analysis of a subset of healthy, currently working females from two studies, controls from the Ontario Women’s Diet and Health case-control study (n = 1611), the ‘population-based sample,’ and the Canadian Study of Diet, Lifestyle and Health cohort study (subset n = 1097), ‘the alumni sample’. The research ethics boards at the University of Alberta, Western University, and the University of Toronto approved the alumni cohort study, and ethics approval for the population-based study was granted by the University of Toronto Research Ethics Board. For both studies, selected participants were given a written consent form, and agreed to participate by returning the consent form, signed, during data collection. For the following study, participant records were anonymized and de-identified prior to analysis. Both studies were originally designed to investigate factors related to cancer risk through self-administered questionnaires mailed to potential participants. The first part of each study survey consists of similar demographic and lifestyle questions, alongside a food frequency questionnaire. Table 1 summarizes the two data sources.

**Table 1:** Description of study datasets.

	Population-based study	Alumni cohort
**Official Study name**	Ontario Women’s Diet and Health Study	Canadian Study of Diet, Lifestyle and Health
**Study Objectives**	To determine lifestyle, socio-demographic, and diet factors related to breast cancer risk in Ontario women.	To determine lifestyle, socio-demographic, molecular markers, and diet factors related to all types of cancer in men and women in Canada.
**Type of Study**	Case-Control.	Cohort.
**Data Collection Period**	June 2002 to April 2003.	1995–9, with a small subset collected in 1992.
**Recruitment Method**	Controls: Random-digit dialing in Ontario.	The alumni societies of the University of Toronto, Alberta, and Western University mailed study questionnaires to their alumni. The small subset collected in 1992 was recruited by contacting known individuals of Canadian Cancer Society volunteers.
**Self-administered questionnaire?**	Yes.	Yes.
**Incentive for survey return**	$5.	None.
**Response Rate**	Controls: 85%	University of Toronto: 17.6%
	Cases: 75%	University of Alberta: 18.9%
		Western University: 10.5%
**Total Sample Size**	N = 6195	N = 34,090
**Subset Sample Size**	Healthy controls: n = 3474	Healthy subset: n = 2170
	Current workers: n = 1611	Current workers: n = 1097
**Shift work question in questionnaire**	What is your current occupation?	What is your usual job or occupation (that is, the job or occupation you have spent the most time in)? Please describe what you actually do/did in this job.

The population-based sample consisted of Ontario females aged 21–73. The healthy controls were selected via random-digit dialing between June 2002 and April 2003 and were offered a $5 incentive for questionnaire return. The response rate for controls was 85% (n = 3474 out of 4102 eligible females).

The alumni sample consisted of Canadian females aged 21–100, recruited through the Universities of Alberta, Toronto, and Western University’s alumni associations between 1995 and 1999, with a small group collected in 1992 by the Canadian Cancer Society (CCS). Volunteers from CCS recruited known contacts into the study and the alumni associations mailed survey packages on behalf of the study investigators to their alumni. Response rates for the Universities of Alberta, Toronto, and Western University were 18.9%, 17.6%, and 10.5% respectively. A response rate for the respondents recruited by CCS could not be determined.

Research ethics approval was obtained for both of the studies from the affiliated universities. For a more detailed description of the datasets see Cotterchio[33] and Rohan[34]. Analyses were carried out on: 1) currently working female controls from the population-based study (n = 1611), and 2) a subset of currently working healthy females from the alumni cohort (n = 1097).

### Measurements

#### Outcomes: Overweight and Obesity

Body mass index (BMI) scores, defined as weight (kg) divided by the square of height (m), were calculated from self-reported current weight and height. The outermost 0.2% of weight and height responses were eliminated to exclude outliers. Weight proportions were determined using the World Health Organization’s classification system with normal/underweight: BMI<25, overweight: BMI = 25 to <30, and obese: BMI≥30.[11]

#### Main Independent Variable: Shift work

Neither study questionnaire directly queried shift work (see Table 1). We determined shift work exposure by linking reported occupation to a shift work probability (*P*_shift work_) value using exposure assessment recently developed from Survey of Labour and Income Dynamics (SLID) 1996 data (N = 12,500), held by Statistics Canada. The SLID is a nationwide survey that captures Canadian labour market information, in flux, by following a panel of 20,000 Canadians every 3 years for 6 years duration.[32]

In the SLID, participants report both their occupation and work schedule. Shift work was defined as regular evening, night, or rotating shifts held for more than 20 hours/week. In exposure assessment, the number and proportion of shift workers in the SLID was tabulated according to sex and both four-digit (specific) and two-digit (broad) standardized occupational codes in line with the National Occupational Classification for Statistics (NOC-S), 2001.[35] The NOC-S 2001 is a framework that classifies over 30,000 occupations in the Canadian labour market into four-tiered hierarchical groupings based on the type of work usually performed. After converting reported occupation in each sample to both four-digit and two-digit NOC-S codes, respondents were linked to female-specific *P*_shift work_ values from the exposure assessment.

**Fig 1:**
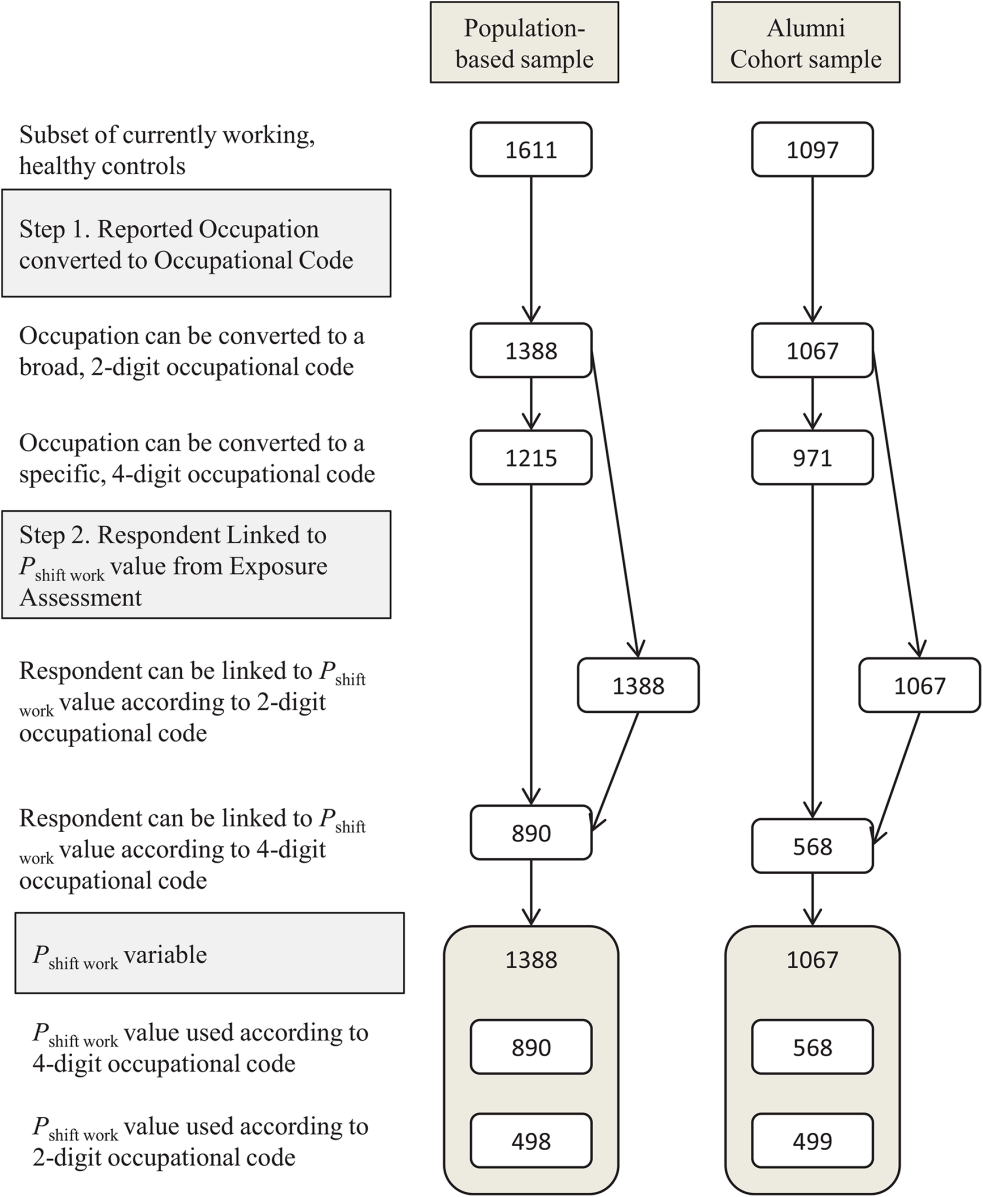
Sample size flow chart for each study sample in accordance with the creation of the shift work variable.

Factors inherent in the questionnaires and in the exposure assessment led to reductions in sample size (See Fig 1). Firstly, due to the open-ended occupational question in both questionnaires, some respondents reported occupations that were too vague for a broad, two-digit NOC-S code (population-based sample: n = 223; alumni cohort: n = 30), or provided an occupation that could be coded at the two-digit level but was too vague for a specific four-digit occupational code (population-based sample: n = 173; alumni cohort: n = 96). In addition, Statistics Canada maintained confidentiality by concealing *P*_shift work_ values if there were fewer than 25 total workers in an occupational code, or, if there were fewer than five shift workers in an occupational code with less than 100 total workers. Therefore, some occupations that were coded at the four-digit level could not be linked to a *P*_shift work_ value based on their specific, four-digit occupational code (population-based sample: n = 325; alumni cohort: n = 403). Of a total of 1611 currently working females in the population-based sample, 890 (55%) were able to receive a specific, four-digit *P*_shift work_ value, whereas 1388 (86%) received a broad, two-digit *P*_shift work_ value. From 1097 currently working females in the alumni cohort, 568 (52%) received a four-digit *P*_shift work_ value, and 1067 (97%) received a two-digit *P*_shift work_ value.

In response to the data suppression, we created a shift work variable which uses *P*_shift work_ values at the specific four-digit level, if available, then refers to the broader two-digit *P*_shift work_ values when the four-digit *P*_shift work_ value is suppressed. We then categorized participants into four *P*_shift work_ exposure levels based on each person’s probability of shift work: near nil (*P*_shift work_<1.5%), low (*P*_shift work_ = 1.5% to <10%), medium (*P*_shift work_ = 10% to <45%), and high (*P*_shift work_ = 45%+). We also added an additional “missing *P*_shift work_” category to account for people whose occupation could not be assigned a *P*_shift-work_ value, as well as former workers (population-based sample n = 1766; alumni cohort n = 1056) as an extra comparison category. In both samples, everyone in the high *P*_shift work_ group received a four-digit, specific *P*_shift-work_ value. Most people in the near nil *P*_shift work_ group also received a four-digit probability of shift work value (83% in the population-based sample, and 98% in the alumni cohort).

#### Confounding Variables

Confounding variables changed the parameter of interest by at least 10.0% and were detected through forward selection. Variables assessed for confounding were age, education (secondary school diploma or less, post-secondary, post-graduate or professional school), race (white, non-white), marital status, smoking in pack years calculated as the average number of cigarettes smoked per day multiplied by the number of years smoked (never, <15, ≥15 pack years), average daily caloric intake (outermost 0.2% excluded), alcohol consumption (never, 1–6, 7+ drinks per week), physical activity (<1 vs. ≥1 time per week), and parity (0, 1, 2–3, ≥4 children).

### Statistical Analyses

All analyses were performed using SAS version 9.2.[36] Frequency and percentage distributions for selected variables were tabulated for each sample dataset. The association between *P*_shift work_ exposure (near nil, low, medium, high, missing) and overweight and obesity was determined through adjusted polytomous logistic regression, expressed as odds ratios (OR). Significance was determined at α = 0.05 (two-tailed).

## Results

Descriptive data is reported in Table 2. In the population-based sample, 30% were overweight, and 19% were obese, and on average, females were overweight (BMI = 25.9, Standard Deviation (SD) = 5.4). In the alumni cohort, females were typically of normal weight (BMI = 24.2, SD = 4.0) with 25% overweight and about 8.5% obese.

**Table 2:** Distribution of selected variables amongst current workers in each study sample.

Study Survey	Population-based sample	Alumni cohort sample
Sample Size	n = 1611	n = 1097
Variable	Frequency (%) or Mean ± S.D.	Frequency (%) or Mean ± S.D.
**BMI**	25.9 ± 5.4	24.2 ± 4.0
**Weight Classification**		
Normal/Underweight		
BMI: <25	813 (50.9)	712 (66.5)
Overweight		
BMI: 25 to <30	477 (29.9)	268 (25.0)
Obese		
BMI: 30+	306 (19.2)	90 (8.4)
**Probability of Shift Work**		
Near Nil: <1.5%	182 (11.3)	260 (23.7)
Low: 1.5 to <10%	660 (41.0)	464 (42.3)
Medium: 1 to <45%	434 (26.9)	274 (25.0)
High: 45%+	112 (7.0)	69 (6.3)
Unattainable	223 (13.8)	30 (2.7)
<Non-Workers>	1766	1056
**Age**	46.4 ± 8.5	49.0 ± 10.2
**Education**		
Secondary school or less	525 (32.6)	29 (2.6)
Some or all post-secondary (college, undergraduate, vocational)	919 (57.1)	760 (69.3)
Graduate or professional degree	165 (10.3)	308 (28.1)
**Race**		
White	1456 (90.4)	1034 (94.4)
Non-white	155 (9.6)	61 (5.6)
**Marital Status**		
Never married	101 (6.3)	154 (15.2)
Married/Common law	1242 (77.1)	811 (79.8)
Divorced or Separated	220 (13.7)	24 (2.4)
Widowed	48 (3.0)	27 (2.7)
**Parity** (# of children)		
0	236 (14.7)	279 (26.9)
1	900 (56.0)	461 (44.5)
2–3	342 (21.3)	206 (19.9)
≥4	128 (8.0)	91 (8.8)
**Smoking** (pack yrs)		
Never	786 (49.4)	634 (59.6)
More than 0, <15	490 (30.8)	315 (29.6)
≥15	314 (19.7)	114 (10.7)
**Calories** (per day)	1767.1 ± 646.2	2157.2 ± 777.2
**Alcohol** (drinks per wk)		
Never	742 (46.4)	180 (16.4)
1–6	549 (34.3)	688 (62.8)
≥ 7	309 (19.3)	227 (20.7)
**Physical Activity** (per wk)		
<1	450 (28.3)	383 (35.9)
≥1	1142 (71.7)	685 (64.1)

In the population-based sample, females were on average 46 years old, and females in the alumni cohort averaged 49 years old. In both studies, most respondents were Caucasian, physically active at least once per week, never smokers, and married. Although the majority in both studies attended post-secondary schooling, a secondary school diploma or less was the highest level of education for one-third of females in the population-based sample, compared to 3% in the alumni cohort. Post-graduate or professional schools were attended by 28% of the alumni cohort, compared to 10% in the population-based sample.

A list of the most common occupations within each *P*_shift work_ category, by sample, is in Table 3. In the population-based sample, most respondents in the near nil *P*_shift work_ group were non-legal or medical secretaries (50%), and administrative officers (21%). In the alumni cohort, the majority in the near-nil *P*_shift work_ group were elementary (50%) and secondary school teachers (35%). In the high *P*_shift work_ group, both studies had a large majority of registered nurses; 72% in the population-based sample, and 96% were nurses in the alumni cohort.

**Table 3:** The top three occupations within each category of the *P*_shift work_ variable.

*P*_shift work_ Group:	Population-based sample	Alumni cohort sample
**Near Nil**	Secretaries, except legal and medical (50%)	Elementary school and kindergarten teachers (50%)
	Administrative officers (21%)	Secondary school teachers (35%)
	Sales, marketing, and advertising managers (4%)	Secretaries, except legal or medical (7%)
**Low**	Clerical occupations (general office clerks, receptionists, accounting clerks, customer service clerks etc.) (24%)	Teachers and professors (34%)
	Teachers and professors (16%)	Lawyers, psychologists, religious ministers, program and policy officers (15%)
	Professional occupations in business and finance (accountants, auditors, bookkeepers, human resource specialists etc.) (8%)	Managers in banking, managers in the arts & social sectors, and school principals (12%)
**Medium**	Retail salespersons and sales clerks (11%)	Professional occupations in health (pharmacists, physiotherapists, physicians etc.) (36%)
	Clerical occupations (shippers & receivers, tellers, postal clerks, etc.) (10%)	Professional occupations in art and culture (e.g. writers, musicians, singers) (25%)
	Technical occupations in health (8%)	Social workers, family counsellors (8%)
**High**	Registered nurses (72%)	Registered nurses (96%)
	Registered nursing assistants (9%)	
	Cleaners and janitors (7%)	
**Missing Job**	-	-
**Former Workers**	n/a	Former occupation missing (27%)
		Teachers and professors (28%)
		Registered nurses (8%)
		Professional occupations in health (7%)

Note: Previously held occupations were not sufficiently queried in the population-based study.

*P_shift work_ = probability of shift work.

### Regression Results

The adjusted polytomous logistic regression results comparing the odds of overweight and obesity according to *P*_shift work_ exposure level (reference = near nil *P*_shift work_) is in Table 4. In the population-based sample, a positive association with obesity was found for females with high *P*_shift work_ exposure (OR: 1.88, 95% CI: 1.01–3.51, *p* = 0.047). In the alumni cohort, no association between *P*_shift work_ exposure and overweight or obesity was detected.

**Table 4:** Adjusted polytomous regression results assessing the odds of overweight and obesity in each sample according to *P*_shift work_.

*P*_shift work_	BMI		Weight proportion based on BMI, n (row %)			Adjusted polytomous regression			
Group	Mean		Normal	Overweight	Obese	Overweight OR		Obese OR	
						(95% CI)	*p*	(95% CI)	*p*
**POPULATION-BASED SAMPLE**									
**NNIL**		25.7	90 (50)	60 (33)	31 (17)	1.0	-	1.0	-
n = 182									
**LOW**		25.9	333 (51)	199 (30)	123 (19)	0.94		1.16	
n = 660						(0.65–1.37)	0.75	(0.73–1.84)	0.52
**MED**		26.1	213 (50)	123 (29)	93 (22)	0.87		1.27	
n = 434						(0.59–1.30)	0.51	(0.79–2.05)	0.33
**HIGH**		26.8	48 (44)	34 (31)	28 (25)	1.15		1.88	
n = 112						(0.66–2.00)	0.62	(1.01–3.51)	0.047
**MISSING**		25.4	129 (58)	61 (28)	31 (14)	0.75		0.71	
n = 223						(0.48–1.18)	0.21	(0.40–1.26)	0.24
**NOT WORKING**		26.7	738 (42)	619 (36)	383 (22)	0.90		1.30	
n = 1766						(0.63–1.30)	0.58	(0.84–2.03)	0.24
**ALUMNI COHORT SAMPLE**									
*P*_shift-work_									
**NNIL**		24.6	159 (63)	69 (27)	25 (10)	1.0	-	1.0	-
n = 260									
**LOW**		24.0	313 (69)	103 (23)	40 (9)	0.71		0.69	
n = 464						(0.48–1.03)	0.071	(0.39–1.21)	0.20
**MED**		24.3	177 (66)	67 (25)	23 (9)	0.84		0.80	
n = 274						(0.56–1.28)	0.42	(0.43–1.49)	0.49
**HIGH**		23.9	46 (69)	19 (28)	2 (3)	0.83		0.14	
n = 69						(0.44–1.56)	0.56	(0.02–1.04)	0.055
**MISSING**		23.9	17 (63)	10 (37)	0 (0)	<analyses removed for this group due to low cell count number>			
n = 30									
**NOT WORKING**		24.3	656 (64)	271 (27)	93 (9)	0.73		0.87	
**n = 1056**						(0.51–1.06)	0.10	(0.50–1.51)	0.63

Population-based sample was adjusted for education and age; Alumni cohort sample was adjusted for smoking, parity, and age.

## Discussion

Our study detected mixed results. In the population-based sample, a high probability of shift work was associated with an increased odds of obesity. In the alumni cohort, a high probability of shift work tended towards a negative association with obesity which was not significant.

Many studies have reported a positive finding in one analysis group, and no association in another.[24,37,38] In regards to the positive finding, one theory is that night and rotating work induce sleep disturbances which lead to increases in weight.[39,40]. Another theory is that shift work perturbs the signals that govern the circadian clock, resulting in out-of-phase relationships between hormones, metabolism and the environment that lead to weight gain.[39]

In the alumni cohort, we did not find a significant association between shift work and obesity. This may have occurred because we did not account for other factors that have been linked to obesity, like workplace stress [41]. However, factors related to a highly educated group of people may have also influenced our findings. Research has demonstrated that broadly, BMI declines as education increases, and those with a university degree experience less weight gain.[42–44] Therefore, the high education in the alumni cohort may have contributed to the low prevalence of obesity in this dataset, which was lower than what was observed in the population-based sample, as well as in Canada around the time of data collection [45]. The low prevalence of obesity may have in turn underpowered our ability to detect differentials in obesity by shift work exposure level in the alumni cohort. Furthermore, other social determinants of health associated with high education which we could not control for, such as better health knowledge, social support, social status, personal health practices, higher income, etc., could have affected our results as well, by inducing a protective factor in the relationship between shift work and weight. Future research should investigate the relationship between these factors in the shift work–obesity paradigm.

Exposure assessment tools and job-exposure matrices have been shown to reliably function as a proxy for actual exposure, at times more accurately than self-reported exposure (i.e. yes/no, never/ever).[46,47] However, our exposure assessment was limited by substantial data suppression. We attempted to rectify this problem by including workers with shift work probabilities based on broader two-digit occupational codes which created a trade-off between the inclusion of more people, and the potential for exposure misclassification bias. However, since respondents with shift work probabilities based on two-digit occupational codes clustered in the low and medium shift work groups, this limitation is relatively minor within the shift work exposure categories of most interest, the near nil and high probability of shift work groups. A subsidiary analysis using just four-digit occupational codes demonstrated the same results (see S1 Fig), with the additional finding in the population-based sample, that workers with medium *P*_shift work_ exposure had an increased odds of obesity (OR: 1.79, 95% CI: 1.05–3.05, *p* = 0.03).

Our study avoided workplace biases by including a range of diverse occupations. However, due to the design of the exposure assessment, job type could not be controlled for, and the same jobs clustered within each shift work category (See Table 2). In both samples, the high probability of shift work group was comprised mostly of registered nurses, 72% in the population-based study, and 96% were nurses in the alumni cohort’s high *P*_shift work_ group. Although shift work is common in the nursing profession, some nurses find work in positions with exclusive daytime hours. It is therefore important to recognize the possibility that by chance, one dataset could have recruited more non-shift working nurses than the other dataset, which would have biased our findings.

Research has suggested that shift work can lead to obesity in nurses, in spite of exercise and a healthy diet.[48] Since our results differed by sample, we conducted an exploratory sub-analysis to investigate obesity amongst nurses alone, versus the group of non-nurses in the population-based study’s high probability of shift work group. The non-nursing group comprised janitors, police officers, bartenders, etc. and together, the prevalence of obesity in this group was 43%, compared to 20% amongst the nurses. After controlling for relevant confounders, however, the positive association was removed for both the group of nurses alone (1.14, 95% CI: 0.55–2.38, *p* = 0.73), and non-nurses (2.25, 95% CI: 0.84–6.05, *p* = 0.11).

Our study detected mixed findings: a positive association in a population-based sample, and no association in a cohort of university alumni. Our data suggests that the relationship between shift work and obesity is multi-faceted and could be susceptible to social determinants of health related to high education, such as income, health knowledge, social support, etc. Therefore, workplace weight reduction programs and policies could be beneficial to shift workers, depending on the unique population in question and the challenges that face that particular population. Future research needs to consider the interaction between factors like education, sleep, workplace stress, shift work duration and the social determinants of health, in order to better understand the intricate relationship between unconventional work hours, metabolism, and weight.

## Supporting Information

S1 Fig Adjusted polytomous regression results assessing the odds of overweight and obesity in each sample according to *P*_shift-work_ exposure ascertained using four-digit occupational codes only. (DOCX)
