# Supplementary material for: Shift Work and Obesity among Canadian Women: A Cross-Sectional Study Using a Novel Exposure Assessment Tool
Source: PLoS One. 2015 Sep 16;10(9):e0137561. doi: 10.1371/journal.pone.0137561 (PMC4573513; doi:10.1371/journal.pone.0137561)
Supplement: S1 Fig — (DOCX) [file pone.0137561.s001.docx]

**Supplement 1.** Adjusted polytomous regression results assessing the odds of overweight and obesity in each sample according to *P*_shift-work_ exposure ascertained using four-digit occupational codes only.

| ***P*_shift work_** | **BMI** | **Weight proportion based on BMI, n (row %)** | | | **Adjusted polytomous regression*** | | | |
| --- | --- | --- | --- | --- | --- | --- | --- | --- |
| **Group** | **Mean** | **Normal** | **Overweight** | **Obese** | **Overweight OR**  (95% CI) | ***p*** | **Obese OR**  (95% CI) | ***p*** |
| **POPULATION-BASED SAMPLE** | | | | | | | | |
|  |  |  |  |  |  | | | |
| **NNIL**  n=151 | 25.5 | 79(53) | 47 (31) | 24 (16) | 1.0 | - | 1.0 | - |
| **LOW**  n=304 | 26.4 | 137(45) | 99 (33) | 68 (22) | 1.23  (0.79-1.93) | 0.36 | 1.64  (0.96-2.82) | 0.073 |
| **MED**  n=323 | 26.7 | 143(45) | 98 (31) | 78 (24) | 1.14  (0.73-1.78) | 0.56 | 1.79  (1.05-3.05) | 0.033 |
| **HIGH**  n=112 | 26.8 | 48 (44) | 34 (31) | 28 (25) | 1.21  (0.68-2.14) | 0.51 | 1.93  (1.00-3.71) | 0.049 |
| **MISSING**  n=721 | 25.4 | 406 (57) | 199 (28) | 108 (15) | 0.84  (0.56-1.26) | 0.40 | 0.88  (0.53-1.46) | 0.63 |
| **NOT WORKING**  n=1766 | 26.7 | 738 (42) | 619 (36) | 383 (22) | 1.01  (0.68-1.50) | 0.94 | 1.52  (0.93-2.47) | 0.09 |
| **ALUMNI COHORT SAMPLE** | | | | | | | | |
| *P*_shift-work_ | |  |  |  |  |  |  |  |
| **NNIL**  n=256 | 24.6 | 156 (63) | 69 (28) | 24 (10) | 1.0 | - | 1.0 | - |
| **LOW**  n=127 | 24.2 | 87 (71) | 24 (20) | 12 (10) | 0.61  (0.35-1.05) | 0.075 | 0.65  (0.29-1.44) | 0.29 |
| **MED**  n=116 | 24.8 | 71 (64) | 29 (26) | 11 (10) | 0.88  (0.51-1.50) | 0.63 | 0.85  (0.38-1.92) | 0.70 |
| **HIGH**  n=69 | 23.9 | 46 (69) | 19 (28) | 2 (3) | 0.81  (0.43-1.52) | 0.51 | 0.14  (0.02-1.04) | 0.055 |
| **MISSING**  n=529 | 24.0 | 352 (68) | 127 (24) | 41 (8) | 0.75  (0.52-1.09) | 0.13 | 0.66  (0.38-1.16) | 0.15 |
| **NOT WORKING**  n=1056 | 24.3 | 656 (64) | 271 (27) | 93 (9) | 0.71  (0.49-1.03) | 0.069 | 0.86  (0.49-1.50) | 0.59 |

* = Population-based sample adjusted for age; Alumni cohort sample adjusted for education, smoking, parity, and age.
